# Stabilization of HIF-1α and HIF-2α, up-regulation of MYCC and accumulation of stabilized p53 constitute hallmarks of CNS-PNET animal model

**DOI:** 10.1371/journal.pone.0173106

**Published:** 2017-03-01

**Authors:** Sergey Malchenko, Simone Treiger Sredni, Yingtao Bi, Naira V. Margaryan, Jerusha Boyineni, Indra Mohanam, Tadanori Tomita, Ramana V. Davuluri, Marcelo B. Soares

**Affiliations:** 1 Department of Cancer Biology & Pharmacology, University of Illinois College of Medicine, Peoria, Illinois, United States of America; 2 Department of Surgery, Division of Pediatric Neurosurgery, Feinberg School of Medicine, Northwestern University, Chicago, Illinois, United States of America; 3 Cancer Biology and Epigenomics Program at the Stanley Manne Children’s Research Institute, Ann & Robert H. Lurie Children’s Hospital of Chicago, Chicago, Illinois, United States of America; 4 Department of Preventive Medicine, Division of Health and Biomedical Informatics, Feinberg School of Medicine, Northwestern University, Chicago, Illinois, United States of America; 5 Abbvie Bioresearch Center, Worcester, Massachusetts, United States; 6 Department of Biochemistry, Robert C. Byrd Health Sciences Center, West Virginia University, Morgantown, West Virginia, United States of America; University of Nebraska Medical Center, UNITED STATES

## Abstract

Recently, we described a new animal model of CNS primitive neuroectodermal tumors (CNS-PNET), which was generated by orthotopic transplantation of human Radial Glial (RG) cells into NOD-SCID mice’s brain sub-ventricular zone. In the current study we conducted comprehensive RNA-Seq analyses to gain insights on the mechanisms underlying tumorigenesis in this mouse model of CNS-PNET. Here we show that the RNA-Seq profiles derived from these tumors cluster with those reported for patients’ PNETs. Moreover, we found that (i) stabilization of HIF-1α and HIF-2α, which are involved in mediation of the hypoxic responses in the majority of cell types, (ii) up-regulation of MYCC, a key onco-protein whose dysregulation occurs in ~70% of human tumors, and (iii) accumulation of stabilized p53, which is commonly altered in human cancers, constitute hallmarks of our tumor model, and might represent the basis for CNS-PNET tumorigenesis in this model. We discuss the possibility that these three events might be interconnected. These results indicate that our model may prove invaluable to uncover the molecular events leading to MYCC and TP53 alterations, which would be of broader interest considering their relevance to many human malignancies. Lastly, this mouse model might prove useful for drug screening targeting MYCC and related members of its protein interaction network.

## Introduction

Embryonal tumors of the central nervous system (CNS) are part of a heterogeneous group of tumors with controversial morphology-based diagnosis, and largely unknown origin [1]. They represent 33% of brain tumors, and affect mainly infants and children younger than 3 years. The group includes (i) medulloblastomas, which arise in the cerebellum, (ii) CNS primitive neuroectodermal tumors (CNS-PNET), which occur in the cerebral hemispheres, in the brain stem and spinal cord, and include CNS neuroblastomas, CNS ganglioneuroblastomas, medulloepitheliomas and ependymoblastomas, and (iii) atypical teratoid rhabdoid tumors (AT/RT) [2].

In this manuscript, supratentorial PNET is referred to as CNS-PNET according to the 2007 WHO CNS tumor classification criteria (ICD-O 9473/3, WHO grade IV). CNS-PNET arises from primitive neuroepithelial cells, and occurs predominantly in children and adolescents. These tumors remain largely refractory to currently available modalities of therapy [2, 3]. Histologically, CNS-PNET is composed mainly of undifferentiated or poorly differentiated neuroepithelial cells with variable amounts of components of divergent differentiation along neural, ependymal and astrocytic lines [2].

Recent molecular studies of large cohorts of embryonal tumors have shed some light to their biology. An integrative genomic analysis of CNS-PNET has categorized these tumors into three distinct subgroups: primitive-neural (group 1), oligo-neural (group 2), and mesenchymal (group 3) [4]. The primitive-neural subgroup often exhibits a younger age of onset (≤4 years) and a higher prevalence in female patients. It has the worst prognosis among all three subgroups. On the other hand, patients with tumors of the mesenchymal subtype present with the highest incidence of metastases at the time of diagnosis [4]. Recently, methylation and gene expression analyses performed for another large series of tumors showed a significant overlap of CNS-PNET with a variety of other pediatric brain tumor types, including high-grade glioma, medulloblastoma and ependymoma [5]. Yet another CNS-PNET study revealed that MYCN or MYCC gene amplifications were present in about half of the cases examined, which was correlated with decreased survival [6]. The above mentioned studies underscore the value of molecular stratification for improved diagnosis and treatment of CNS-PNETs.

Unfortunately, these studies are limited in scope due to multiple factors, including availability of a sufficient number of specimens, tumor heterogeneity, surgical accessibility depending upon tumor location etc. Hence, the development of new animal models of CNS-PNETs is necessary [7] to enable advancement in the field.

Recently, we described an animal model of CNS-PNET that was generated by orthotopic transplantation of human Radial Glial (RG) cells into NOD-SCID mice’s brain sub- ventricular zone [8]. In the current study we explore our CNS-PNET animal model further to get new insights into the onset of tumor formation, and we put forth the hypothesis that hypoxia might play a pivotal role in the development of these tumors.

## Materials and methods

### Ethics statement

All animal-related procedures were approved by the Stanley Manne Children’s Research Institutional Animal Care and Use Committee accredited by the Association for Assessment and Accreditation of Laboratory Animal Care and conformed to the standards of the National Institutes of Health (IACUC protocol #: 2011–09).

### Orthotopic transplantation of RG cells to the Sub-Ventricular Zone (SVZ) of the 3rd ventricle in NOD-SCID mice brain

Transplantations of the LC25-R, LC26-R, and LCAS-R RG cells to the SVZ of 3rd ventricle of the brain of NOD-SCID mice (in average ten mice per each RG cell line) were performed as previously described [8]. Derivation of these RG lines was described previously [8]. Briefly, transplantations of RG cells to target SVZ of 3rd ventricle were performed as follows: a 1.0mm burr hole was made approximately 0.3mm dorsal caudal from the bregma. A 26-gauge needle attached to a 25 μl Hamilton syringe was inserted into the depth of 4.0mm from the skull surface using stereotactic guidance. Five microliters containing ~100,000 of the RG cells were inoculated into the brain over a period of 10 minutes. The respiratory rate and the anesthetic depth of all animals were monitored every 5 minutes after the surgery by laboratory personnel until the animals had fully recovered from the anesthesia. No adverse events were encountered during the post-operative care. All mice were kept in standard animal husbandry with regular diet in barrier facilities and monitored 2–3 times per week, including recording of their body weight. The mice were sacrificed at 4–8 weeks post-inoculation by an i.p. injection of Nembutal Sodium 40–70 mg/kg followed by cervical dislocation, at which time brains were harvested and tumors were resected. The tumor tissues were named LC25-RT, LC26-RT, and LCAS-RT, respectively.

### Total RNA isolation

Total RNA isolation from tumor tissues was performed with the PureZOL RNA isolation reagent (Bio-Rad, Hercules, CA), followed by DNAse treatment (Ambion, Austin, TX), according to the manufacturer's instructions. Purity and integrity of the isolated RNA was assessed on the ND-1000 Spectrophotometer (Thermo Fisher Scientific, Waltham, MA).

### RNA-seq and data analysis

RNA-Seq library construction was performed according to the manufacturer’s instructions for RiboZero selection. The resulting libraries were sequenced using Illumina HiSeq2500. The FastQC software (http://www.bioinformatics.babraham.ac.uk/projects/fastqc/) was applied on raw fastq files to examine the sequence quality. Tophat [9] was used for tag alignment and counts for each gene were computed by means of HTSeq Python package [10], using the annotation of the Ensembl genes and only reads that mapped to exons.

Differential expression analysis on the count data was performed using DESeq2 [11], which is based on a negative binomial distribution and uses shrinkage estimation for the variance of the distribution. As an alternative way of quantifying normalized gene and transcript expression, Fragments Per Kilobase of transcript per Million mapped reads (FPKM) values were also derived using Cufflinks [12] and were furthered normalized by upper quartile normalization (GEO record GSE82102).

The hierarchical clustering was performed using R (function heatmap.2 in “gplots” package). Public RNA-seq data: 10 TCGA GBM and 10 LGG paired-end RNA-seq raw fastq files were downloaded from https://cghub.ucsc.edu/cghub/data/analysis/download. 10 PNET RNA-seq samples were downloaded from SRA archive (SRP032476). All these publicly downloaded samples were analyzed using the same pipeline as for our own samples.

Single nucleotide variant (SNV) and small Indel calling were conducted for both RG and tumor samples. Alignment files generated by Tophat2 were used for SNV detection using SAMtools [13] and Varscan [14] with the following parameters: map quality>15, PHRED quality score>10, coverage>8 reads, P value threshold for calling variant = 0.01 and minimum supporting reads at a position to call variant = 2. We used ANNOVAR [15] for annotation of the called variants and SAMtools view was used to visualize the aligned reads in the region of TP53.

### Real-time PCR

Total RNA isolation was performed as mentioned above. cDNA synthesis and real-time quantitative reverse transcription-polymerase chain reactions (qRT-PCR) were performed as previously described [16]. QuantStudio 7 instrument (Applied Biosystems, USA) along with PowerUP SYBR Green Master Mix (A25742, Fisher) were used according to the manufacturer's instructions. The PCR conditions were as follows: one cycle at 50°C for 2 min, one cycle at 95°C for 10 min, 40 cycles at 95°C for 15 s, 60°C for 1 min, followed by a melting curve from 60°C to 95°C. Primers were designed using the Primer Express program version 1.5 (Applied Biosystems, CA, USA), and obtained from Integrated DNA Technologies (Coralville, IA, USA). Primer sequences were as follows:

TP53

5′ Primer TCAACAAGATGTTTTGCCAACTG

3′ Primer ATGTGCTGTGACTGCTTGTAGATG

P21

5′ Primer TGTCCGTCAGAACCCATGC

3′ Primer AAAGTCGAAGTTCCATCGCTC

100 nM primers for GUSB RNA (RealTimePrimers.com) were used as an endogenous control for each of the cDNA samples. Comparative Ct method was used to analyze the qRT-PCR. In the comparative Cт method the QuantStudio 7 software measures amplification of the gene of interest (target) and of GUSB in each cDNA sample. Measurements are normalized using the endogenous control. The software determines the relative quantity (RQ) of target in each sample by comparing normalized target levels in each sample (LC25-RT, LC26-RT, or LCAS-RT) to normalized target quantity in the reference sample (LC25-R, LC26-R, or LCAS-R).

### Immunohistochemistry

Formalin-fixed paraffin embedded (FFPE) tumor tissue and RG cell pellets generated in our previously published study [8] were used for histological and immunohistochemical analyses. At least two slides (5μm thick) from each FFPE tumor sample were used for the analysis of each antibody presented in this study using standard immunohistochemical methods (see S2 Table).The immunohistochemical panel comprised the following antibodies: Anti-Ki-67 (RM-9106, Rabbit monoclonal, 1:200, Thermo scientific), POU5F1/OCT3/4 (LS-B85, Rabbit polyclonal, 1:300, LSBio), Anti-Nestin (ab105389, Rabbit monoclonal, 1:30, abcam), Anti- Sox2 (ab97959, Rabbit polyclonal, 1:200, abcam), Anti-Vimentin (NBP1-97671, Mouse monoclonal, 1:500, Novus Biologicals), Anti-c-MYC (ab32072, Rabbit monoclonal[Y69], 1:500, abcam), Anti-c-MYC-(Phospho S62) (ab185656, Rabbit monoclonal, 1:500, abcam), Anti-MAX (ab101271, Rabbit polyclonal, 1:1000, abcam), Anti-HIF-1α (ab82832, Rabbit polyclonal, 1:100, abcam), Anti-HIF-2α (ab73895, Rabbit polyclonal, 1:250, abcam), Anti-p53 (LS-B7722, Rabbit polyclonal, 1:200, LSBio), Anti-YB1 (ab12148, Rabbit polyclonal, 1:750, abcam), Anti-MDM2 (LS-C199239, Rabbit polyclonal, 1:100, LS Bio).

### RG cell culturing under hypoxic conditions

RG cells were cultured for 24 days under the same conditions, except for the concentration of O_2_, which was either alternated hypoxic (16 days—5% O_2,_ 4 days—20% O_2,_ 4 days—5% O_2_) or normoxic (20% O_2_). LC26-10R is one of the clones of the LC26-R cell line.

### Western blotting

Whole cell lysates were prepared using M-PER™ Mammalian Protein Extraction Reagent plus protease and phosphatase inhibitors (Thermo Scientific, Rockford, IL). After centrifugation at 13K rpm for 15 minutes at 4°C, the protein concentration of the supernatant was determined using a Pierce™ 660nm Protein Assay Kit (Thermo Scientific, Rockford, IL) and 40μg of total protein loaded per well of a Novex 4–12% NuPAGE Bis-Tris gel (Life Technologies, Carlsbad, CA). After electrophoresis, the proteins were transblotted onto a nitrocellulose blotting membrane (Bio-Rad Laboratories, Hercules, CA) and the proteins detected using Anti-HIF-2α (ab73895, Rabbit polyclonal, 1:250, abcam), Anti-POU5F1/OCT3/4 (LS-B85, Rabbit polyclonal, 1:300, LSBio), Anti- c-MYC-(Phospho S62) (ab185656, Rabbit monoclonal, 1:500, abcam), Anti- Sox2 (ab97959, Rabbit polyclonal, 1:200, abcam), goat anti-rabbit antibody linked to horseradish peroxidase (sc2030 Santa Cruz, Dallas, TX) and ECL substrate (Western Bright ECL, Advansta,Menlo Park, CA). The blot was then stripped and probed for GAPDH or Beta-actin protein using a mouse monoclonal antibody (GAPDH: SC365062, Santa Cruz, Dallas, TX, Beta-actin: MA5-15739, ThermoFisher Scientific, Waltham, MA), goat anti-mouse antibody linked to horseradish peroxidase (sc2005 Santa Cruz, Dallas, TX) and ECL substrate. Protein levels were estimated by densitometry using ImageJ software and the differences in HIF-2a, Oct3/4, c-MYC-(Phospho S62) and Sox2 proteins calculated after correcting for protein loaded per lane using the GAPDH or Beta-actin protein control.

## Results

### RNA-Seq profiles of tumors from the CNS-PNET animal model cluster with those derived from patients’ CNS-PNETs

**Fig 1:**
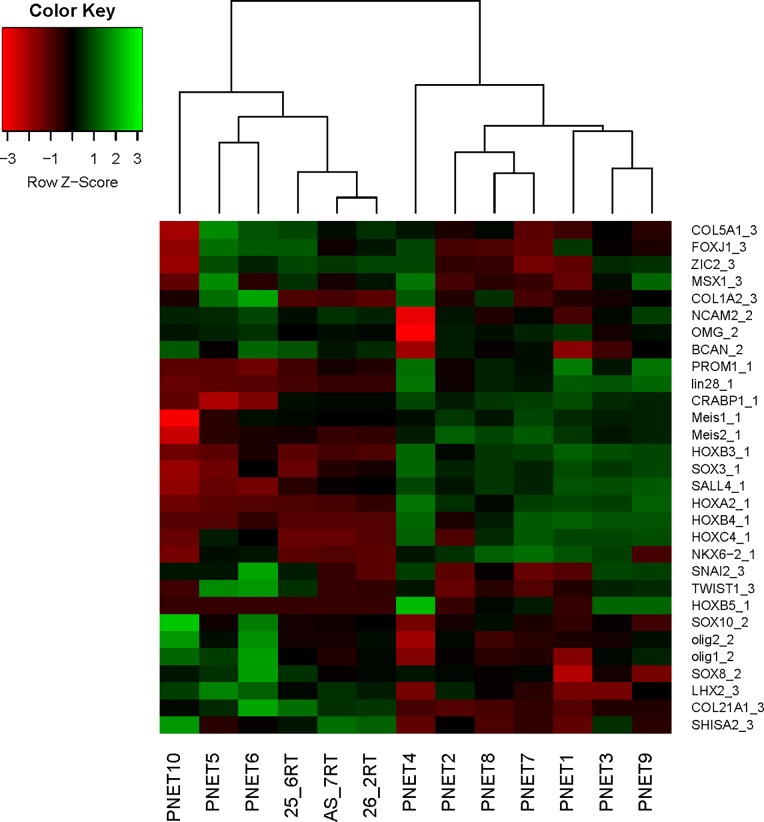
Supervised hierarchical clustering analysis of our CNS-PNET model tumors (molecular markers). List of molecular markers of CNS -PNET according to Picard’s classification. Gene symbol_1—primitive-neural (group 1) marker, Gene symbol_2—oligo-neural (group 2) marker, Gene sybmol_3—mesenchymal (group 3) marker.

**Fig 2:**
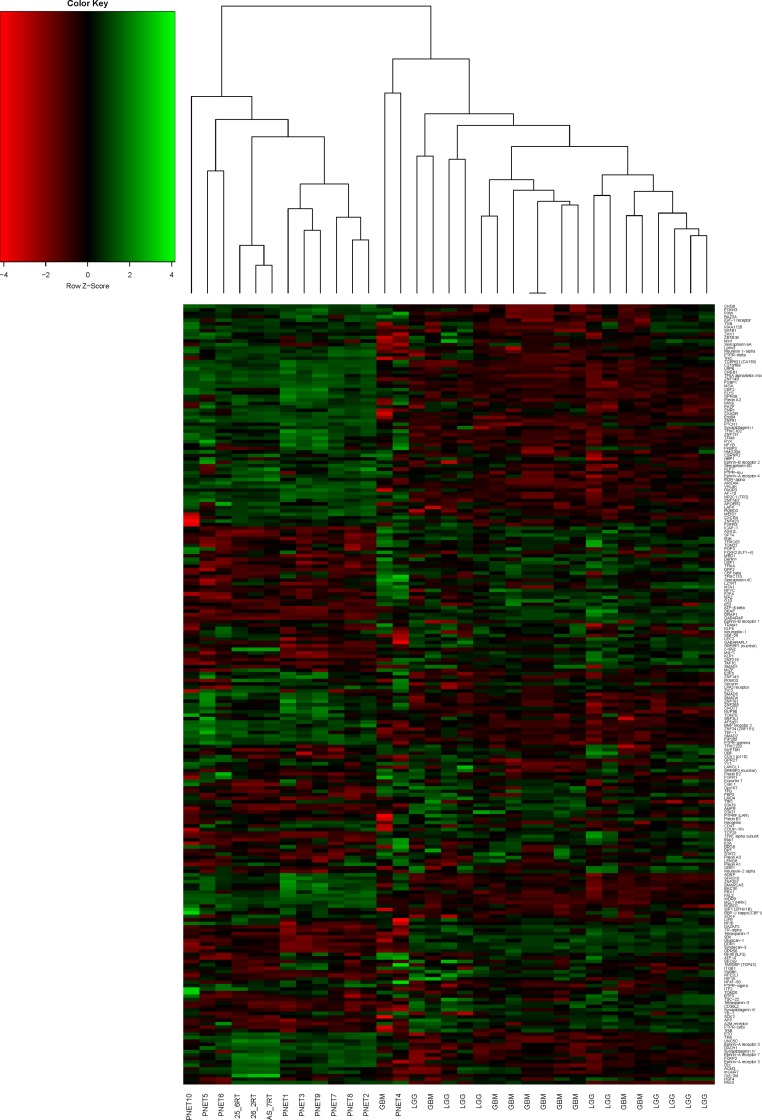
Supervised hierarchical clustering analysis of our CNS-PNET model tumors (transcription factors and receptors). List of the 225 transcription factors and receptors (S1 Table), which are consistently expressed in our tumors at intermediate or high level of expression (similar or higher than average house-keeping gene expression level).

Histopathological analyses of the tumors from our model revealed hallmarks of neuroectodermal origin that are characteristic of CNS PNET [8]. We sought to validate this observation by applying unsupervised hierarchical clustering of RNA-seq data from three of our CNS-PNET model tumors (LC25-RT, LC26-RT, and LCAS-RT) along with ten CNS-PNETs, ten glioblastomas (GBM) and ten low-grade gliomas (LGG), from publicly available RNA-seq data (see Materials and Methods). We also applied a supervised hierarchical clustering analysis of the same dataset using a list of 225 TFs and receptors, which are consistently expressed in our tumors at intermediate or high levels (similar to or higher than the average level of expression observed for house-keeping genes; S1 Table). Additionally, we used a list of genes that have been identified as predictive markers of PNET according to Picard’s molecular classification [4]. Based on the unsupervised hierarchical clustering analysis, our tumors clustered with the CNS-PNET group (S1 Fig). Applying Picard's molecular signature, our tumors clustered with the mesenchymal and oligo-neural subtypes of CNS-PNET (Fig 1). Interestingly, the clustering analysis of TFs and receptors revealed that our tumors are closest to the primitive-neural subgroup of CNS-PNETs (samples 1, 2, 3, 7, 8, 9; see Fig 1 and Fig 2).

### The CNS-PNET model tumors are enriched with markers of NSC self-renewal

There is evidence that brain tumorigenesis can be driven by alterations occurring in neural stem cells (NSCs) that render them transformed to brain tumor-initiating cells (BTICs) [17, 18]. In particular, presumably transformed RG cells have been considered to function as putative BTICs in ependymomas [18]. Histologically, divergent differentiation of CNS-PNET, which is reflected by variable amounts of neuronal, ependymal, and glial cellular components within the tumor, may be interpreted as indicative of the presence of both NSCs and BTICs in these tumors, and that the latter may be responsible for its development [2]. Notwithstanding all supportive evidence, however, it should be underscored that the presence of cells with tumor-initiating properties does not necessarily imply causality for tumorigenesis, nor does it exclude the role of other cells. The existing data simply serve to demonstrate that such tumors indeed harbor cells that have the capability of initiating tumors.

**Fig 3:**
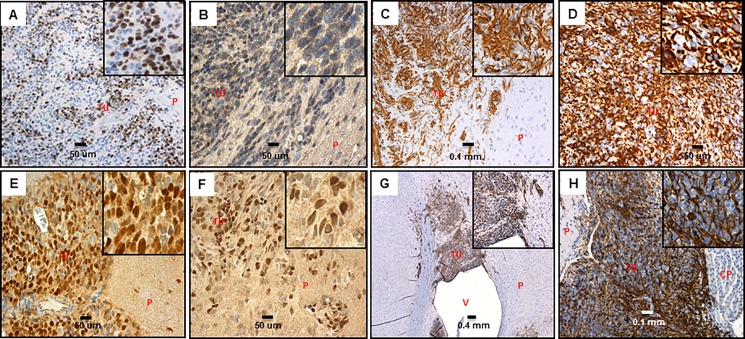
BTIC markers in our CNS-PNET model tumors. A: Ki67, 20X, insert- 5X digital (LCAS-R-12 weeks post-injection) B: Oct3/4, 40x, insert- 5X digital (LCAS-R-12 weeks post-injection) C: Nestin 20x, insert- 5X digital (LCAS-R-12 weeks post-injection) D: Nestin 40x, insert- 5X digital (LCAS-R-12 weeks post-injection) E: Sox2 40X, insert- 5X digital, (LC26-R-12 weeks post-injection) F: Sox2 40X, insert- 5X digital (LCAS-R-12 weeks post-injection) G: Vimentin 5X, insert- 5X digital (LCAS-R-12 weeks post-injection) H: Vimentin 20X, insert- 5X digital (LC26-R-12 weeks post-injection) TU-tumor; P-parenchyma; V-ventricle; CP-choroid plexus.

The initiation of tumors in our CNS-PNET model by orthotopic transplantation of RG cells suggested the presence of BTICs amidst the RG cell population, which would be responsible for the onset of tumor formation. Using our CNS-PNET model, we validated the expression of the NSC self-renewal markers SOX2 (S1 and S2 Tables, Fig 3E and 3F), Nestin, and Vimentin, which are also considered as BTIC markers [8, 16, 17, 19–23], including in CNS-PNET [7, 24] (S2 Table, Fig 3C, 3D, 3G and 3H). Remarkably, Oct3/4 (POU5F1), which was recently described as a new BTIC marker [19], is also expressed in our tumor model (Fig 3B, S1 and S2 Tables). It is conceivable that, in our model, the expression of markers associated with self-renewal of NSC/BTIC in tumor cells might be responsible for maintenance of the primitive neuroectodermal phenotype [8]. It is also noteworthy that the presence of these markers in the majority of the tumor cells (Fig 3, S2 Table) correlates with the rapid progression to large, aggressively disseminating undifferentiated tumors documented in our model [8]. However, as aforementioned, the mere presence of these markers cannot be deemed sufficient to render them functional BTICs. Such possibility will be the subject of future investigation.

### HIF-1α and 2α stabilization, up-regulation of OCT3/4 and MYCC provide a plausible mechanism for tumorigenesis in our CNS-PNET model

We hypothesize that hypoxia is one of the micro-environmental factors involved in the onset of tumorigenesis in our model [8], as it is a critical component of stem cell niche function, keeping the undifferentiated phenotype of stem/precursor cells [25, 26]. Since our RG cells represent the early stages of primitive neuro-ectoderm differentiation [16], and the stem cells that normally reside in the 3^rd^ sub-ventricular zone correspond to adult NSCs, it is conceivable that while protective for the adult NSC, hypoxia might trigger tumorigenesis of the orthotopically transplanted RG cells that are at a significantly earlier stage in ontogenesis. Low oxygen level could lead to stabilization of HIF-1α and 2α in parental RG cells. This, in turn, would activate BTIC marker OCT3/4, a direct target of HIF-2 α, along with BTIC marker SOX2, and the critical pluripotent stem cell inducer–MYCC, an onco-protein whose increased expression plays a central role in multiple aspects of tumor cell biology [25–32]. Interestingly, HIF-1 α, which is involved in mediation of the hypoxic responses in the majority of cell types, is expressed in our tumor model along with SOX2 and OCT3/4 (S1 Table). Therefore, we reason that altogether, these events could provide a plausible mechanism for tumorigenesis in our model.

**Fig 4:**
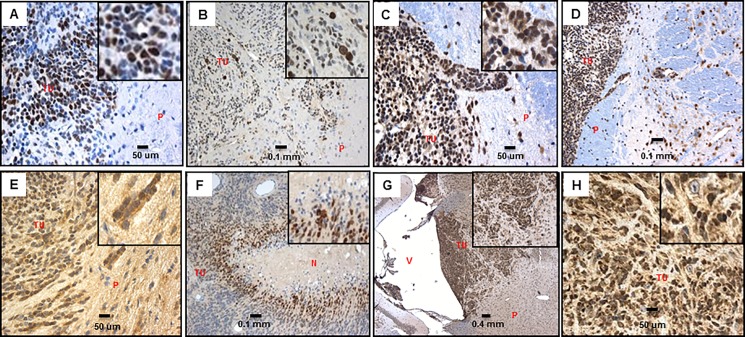
cMYC, c-MYC-(Phospho S62), HIF-1α, HIF-2α in our CNS-PNET model tumors. A: cMYC 40X, insert- 5X digital (LCAS-R-12 weeks post-injection) B: c-MYC-(Phospho S62) 20X, insert- 5X digital (LCAS-R-12 weeks post-injection) C: MAX 40X, insert- 5X digital (LCAS-R-12 weeks post-injection) D: MAX 20X (LCAS-R-12 weeks post-injection) E: HIF-1α 40X, insert- 5X digital (LCAS-R-12 weeks post-injection) F: HIF-1α 20X, insert- 5X digital (LCAS-R-12 weeks post-injection) G: HIF-2α 5X, insert- 5X digital (LCAS-R-12 weeks post-injection) H: HIF-2α 40X, insert- 5X digital (LCAS-R-12 weeks post-injection) TU-tumor; P-parenchyma; V-ventricle; N-necrosis.

To begin to investigate our hypothesis we tested if HIF-1 α and HIF-2 α proteins, which are known to be degraded in normoxic conditions [33], are expressed in the tumor cells. Indeed, immunostaining revealed that the majority of tumor cells exhibit nuclear expression of HIF-1 α and HIF-2 α (Fig 4E–4H, S2 Table), along with SOX2 and OCT3/4, as stated above.

Remarkably, we also found MYCC as being highly expressed in our tumors (Fig 4A, S1 and S2 Tables). In adult tissues, MYCC expression is usually low and restricted to cells with proliferative potential [34]. To activate or repress transcription consistently, MYCC has to be stabilized by phosphorylation at the N-terminus of Ser 62, which is known to protect the MYC protein from proteasomal degradation [35]. The stabilized form of MYCC is also highly expressed in our tumors (Fig 4B, S2 Table). Moreover, MYCC has to heterodimerize with a partner protein, MYC-associated protein X (MAX) [36], which is also abundantly expressed in our model (Fig 4C and 4D, S1 and S2 Tables).

**Fig 5:**
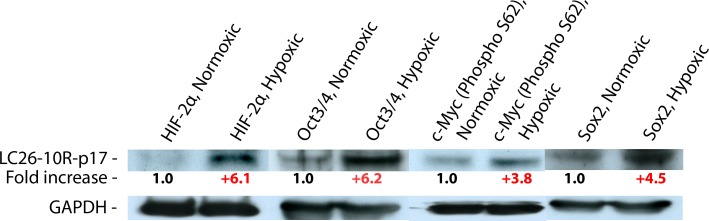
Western blot. Protein quantitative difference between the RG cells grown for 24 days in normoxic versus hypoxic conditions. The quantitative differences of the proteins calculated after the correction for actual protein loaded per lane using the GAPDH protein control.

Next, we tested the response of the RG cells to a prolonged exposure to low oxygen conditions, monitoring the expression level of HIF-2 α, OCT3/4, SOX2, and c-MYC-(Phospho S62) by western blot, using GAPDH or Beta-actin proteins as endogenous controls. Consistent with the aforementioned hypothesis, all these proteins were up-regulated after exposure to hypoxia (Fig 5, S2 Fig).

### Accumulation of stabilized p53 is observed in our CNS-PNET tumor model

Hypoxic microenvironment has been implicated in genetic instability [37, 38]. It increases the frequency of mutations in cell culture as well as in animal tumor models [39]. Consequently, hypoxia could inactivate the function of the key tumor-suppressor gene TP53, which would reduce hypoxia-induced cell death and allow the survival of cells with cumulative DNA damage [40]. Mutations of the TP53 gene are detected in many different types of human cancer [41–43], with the majority of them being missense mutations within the DNA-binding core domain [44]. However, the pathways responsible for these molecular alterations have yet to be identified.

**Fig 6:**
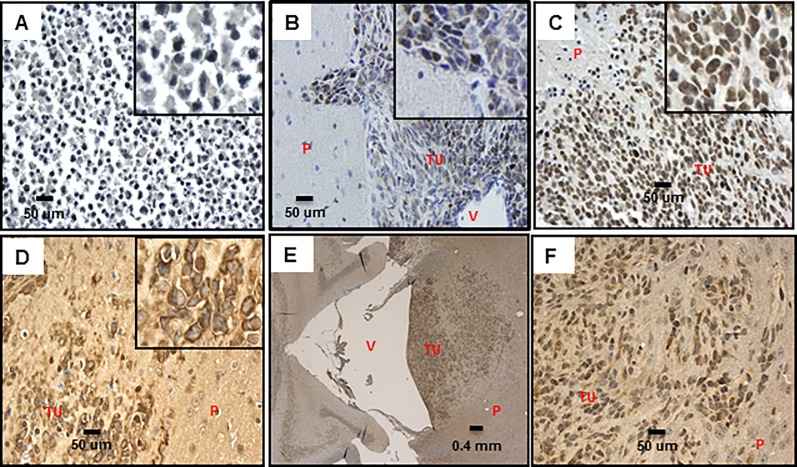
Expression of TP53, YB1 and MDM2 in our CNS-PNET model tumors A. TP53 40x, insert- 5X digital (LCAS-R) B: TP53 40x, insert- 5X digital (LCAS-R-12 weeks post-injection) C: TP53 40x, insert- 5X digital (LC26-R-12 weeks post-injection) D: YB-1 40X, insert- 5X digital (LCAS-R-12 weeks post-injection) E: MDM2 5x, F: MDM2 40x, (LCAS-R-12 weeks post-injection) TU-tumor; P-parenchyma; V-ventricle.

Remarkably, p53 is overexpressed in our model (Fig 6B and 6C, S1 and S2 Tables) with more than 90% of tumor cells showing nuclear expression of the protein. In contrast, it is expressed in less than 10% of the parental RG cells (Fig 6A). This pattern of p53 immunoexpression in the tumors is indicative of the presence of mutant p53 protein. As wild-type p53 protein is relatively unstable it is virtually undetectable by immunohistochemistry, while the mutant p53 becomes stabilized, and accumulates in the nucleus, thereby making its detection by immunohistochemistry possible [45]. Surprisingly, comparative sequence analysis of RNA-Seq data derived from TP53 in the parental RG and tumor cells in our model did not reveal any SNVs or indels (data not shown, see Material and Methods). It is conceivable, however, that the documented stabilization of p53 might be due to an aberrant posttranslational modification, which is known to suppress p53 functional activities, including growth arrest and transcriptional activation of target genes [46], as the stabilization of p53 is depicted in actively invading tumor cells (Fig 6B and 6C digital inserts). Notably, we found the transcription factor YB1 being expressed at high levels in our tumor model (Fig 6D, S1 and S2 Tables). YB1 expression in the cytoplasm of tumor cells is consistent with the functional inactivation of TP53, as nuclear translocation of YB1 would require expression of wild-type p53 [47, 48]. YB1 is an essential marker of tumorigenesis as its expression significantly correlates with tumor stage and patient prognosis for many human tumors, including glioblastoma and medulloblastoma [49–53]. The aberrant modification of p53 protein might also render resistance to MDM2 mediated degradation [46], despite the elevated level of MDM2 in the tumor tissue (Fig 6E and 6F, S2 Table). MDM2 is involved in ubiquitination and proteasome degradation of wild-type p53 protein, and considered as one of the key regulators of p53 stability [46]. Of note, the mRNA level of TP53 is reduced in the tumor (S3A Fig), along with the mRNA level of P21 (S3B Fig)–a direct target of active p53 [48], which might indicate the functional integrity of MDM2 as it is known to block TP53 transcription [54, 55]. Nevertheless, at this point we can’t unequivocally prove the functional inactivation of the stabilized p53, which will be the subject of a follow up study.

## Discussion

As PNET transcriptome profiles from recent studies exhibit a significant overlap with those observed for a number of brain tumor types [5], it is conceivable that cell origin or onset of tumorigenesis are also shared in these tumors, which make our model potentially relevant to a broad range of brain tumors, including a variety of embryonal tumors, beyond CNS-PNET.

The fact that tumors in our model can be consistently generated in only 4–8 weeks, rapidly progressing to large, aggressively disseminating tumors point to the robustness of the molecular mechanisms underlying tumorigenesis in our model. Such a behavior may suggest the occurrence of rather few discrete alterations of major functional significance, most likely of epigenetic nature [56]. Admittedly highly speculative, one possible scenario may be that the hypoxic micro-environment of the 3^rd^ sub-ventricular zone triggers tumorigenic epigenetic alterations in our RG cells, whose transcription and epigenetic landscapes represent the early stages of primitive neuro-ectoderm differentiation [15]. The presence of RG—BTIC markers such as SOX2, Vimentin, Nestin, and of BTIC marker OCT3/4 also suggest that our CNS-PNETs might arise directly from the orthotopically transplanted RG, or from its early progeny, which, based on our hypothesis, would undergo transformation to become BTICs, thus initiating tumorigenesis. It is also conceivable that the MYCC onco-protein plays a central role in this process.

MYCC is evolutionarily conserved [57], and its role as a signal transducer linking the extracellular and intracellular domains may track back to single-cell organisms [58]. It is a highly multifunctional transcription factor [59–61], which acts as a general amplifier in the regions of open chromatin at the time of MYCC activation [62]. Deregulated expression of MYCC in cancer commonly occurs through constitutive activation of upstream signaling pathways. [63].

Overexpression of MYCC enhances HIF-1 α accumulation under hypoxic conditions [64]. Moreover, the dysregulated expression of MYCC synergizes with HIFs to form the tumor metabolic phenotype that is described as aerobic glycolysis [65], and to promote MYCC -induced anchorage-independent growth and cell proliferation [64]. MYCC is essential not only for tumor initiation and progression but also for tumor maintenance [66–72]. As with HIFs, the dysregulated expression of MYCC could also synergize with the loss of the tumor suppressor TP53, which in turn would induce cellular proliferation and tumorigenesis [73], particularly in NSCs [74]. A recently published study revealed significant association of TP53 mutation and MYC gene family up-regulation at relapse in medulloblastoma patients [75].

Despite the fact that altered expression of the MYCC oncogene occurs in ~70% of human tumors, the specific mechanism that affects MYCC in each tumor type is still largely unknown [76, 32]. Moreover, a comprehensive assessment of the MYCC target genes in different brain tumors is also missing. Such a comprehensive identification of MYCC target genes might constitute the basis for a better understanding of the mechanisms underlying MYCC-driven tumorigenesis, and for development of new therapeutic strategies. Our model might be exploited to facilitate such analyses and to decode PNET’s specific mechanism of MYCC dysregulation. Lastly, it might also be used to validate experimental drugs targeting MYCC, which are likely to aid in combined therapy regimens [77–79].

Fundamental questions remain regarding the mechanisms by which hypoxia induces genetic alterations (such as TP53 mutation), controls cell proliferation, and contributes to the altered metabolic phenotype of cancer cells. Furthermore, it remains to be determined if hypoxia is indeed responsible for triggering tumorigenesis in our model, which will be the object of future investigations. These studies are likely to prove relevant to the broad range of tumors that are affected by hypoxia.

## Conclusions

In conclusion, we showed that the presence of stabilized HIF-1 α and HIF-2 α, up-regulation of MYCC, and accumulation of stabilized p53 constitute hallmarks of CNS-PNET in our model, and we speculate that such alterations might represent the basis for tumorigenesis in this tumor model. We also put forth the hypothesis that these three events might be interdependent, and that hypoxia might be one of the initiating micro- environmental factors underlying tumorigenesis in our CNS-PNET model.

## Supporting information

S1 Fig A clustering analysis of RNA seq. data from the three of our CNS-PNET model tumors of different origin, along with ten PNETs, ten glioblastomas (GBM) and ten low grade gliomas (LGG) from publicly available RNA seq. domains. (TIF)

S2 Fig Western blot. Protein quantitative difference (triplicate) between the RG cells grown for 24 days in normoxic versus hypoxic conditions. The quantitative differences of the proteins calculated after the correction for actual protein loaded per lane using the Beta-actin protein control. OCT3/4 protein quantitative difference was meagured as a duplicate due to unspecific background signal in the area of the protein band in normoxia conditions from one of the experiments.
(TIF)

S3 Fig Real-time PCR. A: TP53 relative quantity (RQ) in LCAS-RT compared to LCAS-R, B: P21 relative quantity (RQ) in LCAS-RT compared to LCAS-R.
(TIF)

S1 Table Table of TF and receptors: 225 transcription factors and receptors, which were consistently expressed in our tumors at intermediate or high level of expression (similar or higher than average house-keeping gene expression level). TF-Transcription Factor.
(DOCX)

S2 Table Formalin-fixed paraffin embedded (FFPE) tumor tissue used for the immunohistochemical analyses: X indicates the FFPE tumor tissue, which was analyzed with the Abs presented in the study. (DOCX)
