# Supplementary figures and images for: Stabilization of HIF-1α and HIF-2α, up-regulation of MYCC and accumulation of stabilized p53 constitute hallmarks of CNS-PNET animal model
Source: PLoS One. 2017 Mar 1;12(3):e0173106. doi: 10.1371/journal.pone.0173106 (PMC5332108; doi:10.1371/journal.pone.0173106)

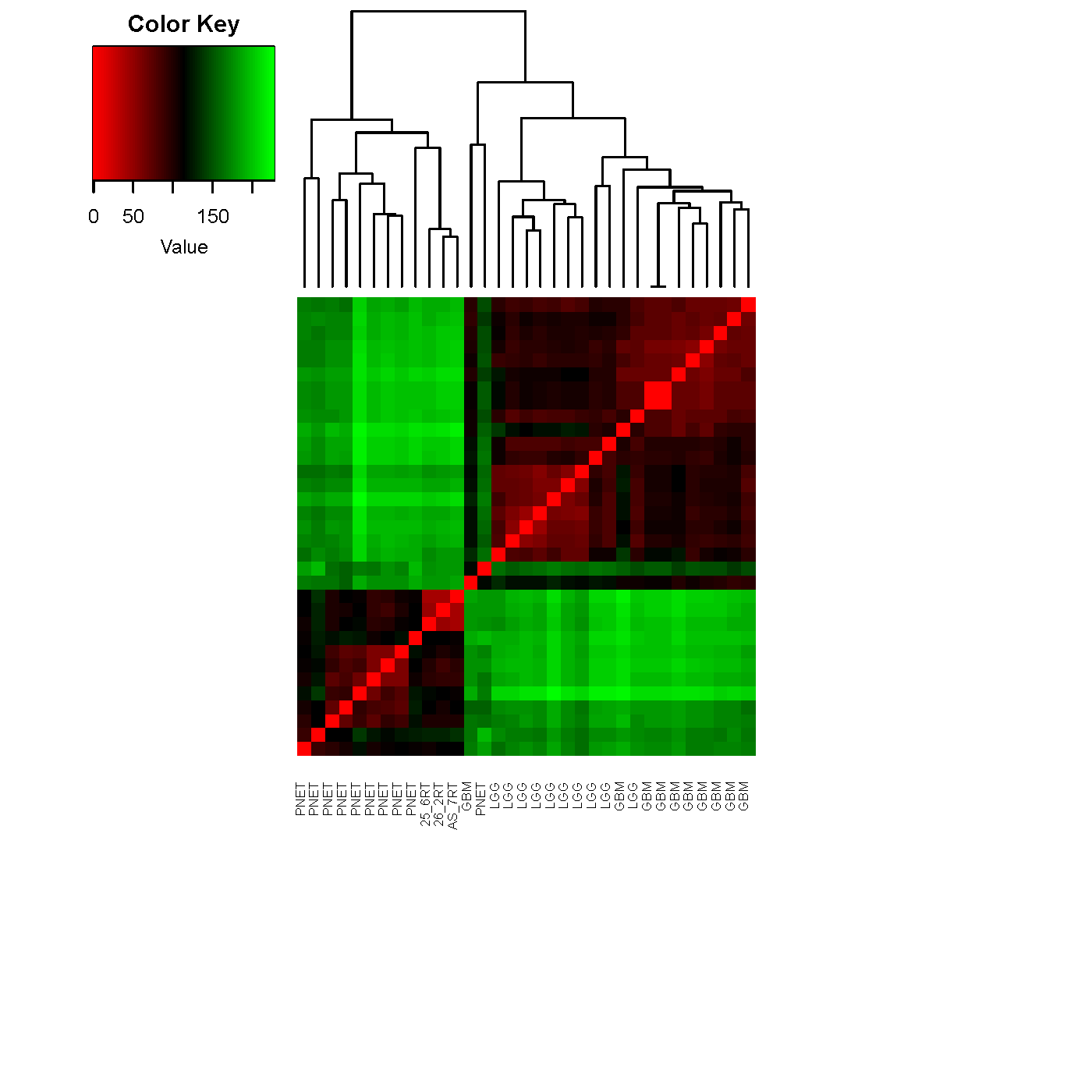

Supplement: S1 Fig — (TIF) [file pone.0173106.s001.tif]

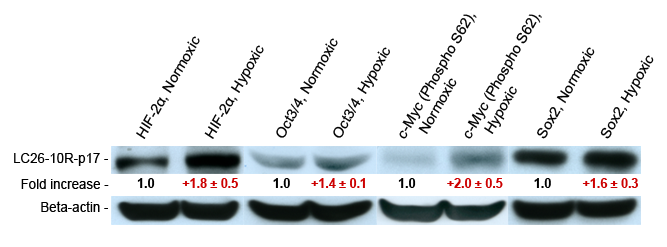

Supplement: S2 Fig — Protein quantitative difference (triplicate) between the RG cells grown for 24 days in normoxic versus hypoxic conditions. The quantitative differences of the proteins calculated after the correction for actual protein loaded per lane using the Beta-actin protein control. OCT3/4 protein quantitative difference was meagured as a duplicate due to unspecific background signal in the area of the protein band in normoxia conditions from one of the experiments. (TIF) [file pone.0173106.s002.tif]

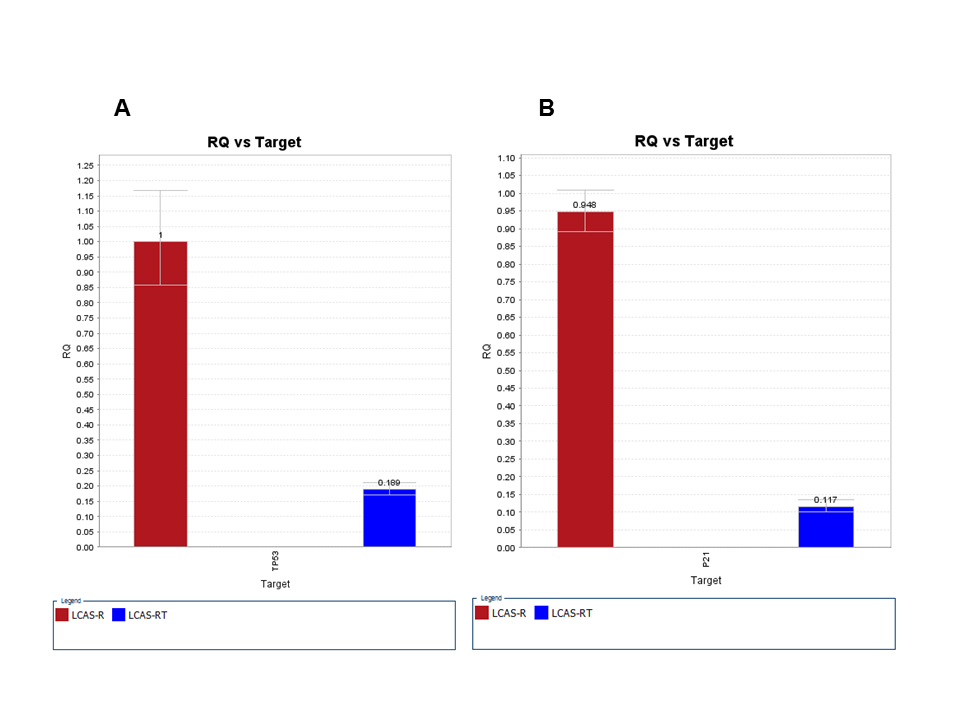

Supplement: S3 Fig — A: TP53 relative quantity (RQ) in LCAS-RT compared to LCAS-R, B: P21 relative quantity (RQ) in LCAS-RT compared to LCAS-R. (TIF) [file pone.0173106.s003.tif]
