# Supplementary material for: Stabilization of HIF-1α and HIF-2α, up-regulation of MYCC and accumulation of stabilized p53 constitute hallmarks of CNS-PNET animal model
Source: PLoS One. 2017 Mar 1;12(3):e0173106. doi: 10.1371/journal.pone.0173106 (PMC5332108; doi:10.1371/journal.pone.0173106)
Supplement: S1 Table — TF-Transcription Factor. (DOCX) [file pone.0173106.s004.docx]

**S1 Table**

| **Ensembl** | **Gene symbol** | **Function** |
| --- | --- | --- |
| ENSG00000167232 | ZNF91 | TF |
| ENSG00000136527 | SFRS10 | TF |
| **ENSG00000065978** | **YB-1** | **TF** |
| ENSG00000145741 | BTF3 | TF |
| **ENSG00000136997** | **MYCC** | **TF** |
| ENSG00000196628 | ITF2 | TF |
| ENSG00000162613 | FUBP1 | TF |
| ENSG00000132274 | Staf-50 | TF |
| ENSG00000007372 | PAX6 | TF |
| ENSG00000010244 | ZNF207 | TF |
| ENSG00000129351 | NFAT-90 | TF |
| ENSG00000102804 | TSC-22 | TF |
| ENSG00000082641 | NFE2L1 | TF |
| ENSG00000143995 | MEIS1 | TF |
| ENSG00000141002 | TCF25 | TF |
| ENSG00000141646 | SMAD4 | TF |
| ENSG00000079102 | ETO | TF |
| ENSG00000143621 | NF45 (ILF2) | TF |
| ENSG00000135903 | PAX3 | TF |
| ENSG00000169554 | SIP1 (ZFHX1B) | TF |
| ENSG00000171634 | FALZ | TF |
| ENSG00000147862 | NFIB | TF |
| ENSG00000175745 | COUP-TFI | TF |
| ENSG00000175387 | SMAD2 | TF |
| ENSG00000120948 | TARDBP (TDP43) | TF |
| **ENSG00000100644** | **HIF1A** | **TF** |
| ENSG00000124766 | SOX4 | TF |
| ENSG00000103495 | MAZ | TF |
| ENSG00000185630 | PBX1 | TF |
| ENSG00000128272 | ATF-4 | TF |
| ENSG00000113649 | TCERG1 (CA150) | TF |
| ENSG00000043355 | ZIC2 | TF |
| ENSG00000119950 | Mxi1 | TF |
| ENSG00000107164 | FBP3 | TF |
| ENSG00000182979 | MTA1 | TF |
| ENSG00000100888 | CHD8 | TF |
| ENSG00000122034 | TFIIIA | TF |
| ENSG00000112983 | BRD8 | TF |
| ENSG00000198911 | SREBP2 (nuclear) | TF |
| ENSG00000106245 | G10 | TF |
| ENSG00000099949 | LZTR1 | TF |
| ENSG00000069667 | ROR-alpha | TF |
| ENSG00000183283 | DAZAP2 | TF |
| ENSG00000185658 | WDR9 | TF |
| ENSG00000128573 | FOXP2 | TF |
| ENSG00000100941 | Pinin | TF |
| ENSG00000118260 | CREB1 | TF |
| ENSG00000159086 | C21orf66 | TF |
| ENSG00000177030 | DEAF | TF |
| ENSG00000113658 | SMAD5 | TF |
| ENSG00000126351 | TR-alpha | TF |
| ENSG00000168214 | RBP-J kappa (CBF1) | TF |
| ENSG00000163848 | ZNF148 | TF |
| **ENSG00000204531** | **OCT3/4** | **TF** |
| **ENSG00000125952** | **Max** | **TF** |
| ENSG00000198791 | CNOT7 | TF |
| ENSG00000170581 | STAT2 | TF |
| ENSG00000153147 | SMARCA5 | TF |
| ENSG00000213246 | SPT4 | TF |
| ENSG00000118058 | MLL1 (HRX) | TF |
| ENSG00000165804 | ZNF219 | TF |
| ENSG00000110713 | NUP98 | TF |
| ENSG00000119041 | TFIIIC102 | TF |
| ENSG00000053254 | FOXN3 | TF |
| ENSG00000165659 | DACH1 | TF |
| ENSG00000187079 | TEF-1 | TF |
| ENSG00000105856 | HBP1 | TF |
| ENSG00000172466 | ZNF24 (ZNF191) | TF |
| ENSG00000120798 | NR2C1 (TR2) | TF |
| ENSG00000182568 | SATB1 | TF |
| ENSG00000108312 | UBF | TF |
| ENSG00000175550 | DRAP1 | TF |
| **ENSG00000141510** | **TP53** | **TF** |
| ENSG00000141644 | MBD1 | TF |
| ENSG00000132485 | ZNF265 | TF |
| ENSG00000172262 | ZNF131 | TF |
| ENSG00000101126 | ADNP | TF |
| ENSG00000023287 | FIP200 | TF |
| ENSG00000072364 | AF5q31 | TF |
| ENSG00000140382 | HMG20A | TF |
| ENSG00000198176 | DP1 | TF |
| ENSG00000066136 | NFYC | TF |
| ENSG00000136451 | ZNF161 | TF |
| ENSG00000177311 | ZBTB38 | TF |
| ENSG00000120837 | NFYB | TF |
| ENSG00000077235 | TFIIIC220 | TF |
| ENSG00000072310 | SREBP1 (nuclear) | TF |
| **ENSG00000181449** | **SOX2** | **TF** |
| ENSG00000158773 | USF1 | TF |
| ENSG00000174197 | MGA | TF |
| ENSG00000102935 | ZNF423 | TF |
| ENSG00000115415 | STAT1 | TF |
| ENSG00000168610 | STAT3 | TF |
| ENSG00000198795 | EHZF | TF |
| ENSG00000100811 | YY1 | TF |
| ENSG00000071564 | E2A | TF |
| ENSG00000170515 | Ebp1 | TF |
| ENSG00000144218 | LAF4 | TF |
| ENSG00000076108 | BAZ2A | TF |
| ENSG00000125651 | TFIIF, alpha subunit | TF |
| ENSG00000116809 | Miz-1 | TF |
| ENSG00000164611 | Securin | TF |
| ENSG00000213676 | ATF-6 beta | TF |
| ENSG00000118263 | KLF7 | TF |
| ENSG00000102878 | HSF4 | TF |
| ENSG00000115207 | TFIIIC110 | TF |
| ENSG00000078403 | AF-10 | TF |
| ENSG00000108064 | TFAM | TF |
| ENSG00000143013 | LMO4 | TF |
| ENSG00000090971 | KLP1 | TF |
| ENSG00000115816 | CBF2 | TF |
| ENSG00000170365 | SMAD1 | TF |
| ENSG00000009954 | BAZ1B | TF |
| ENSG00000067082 | KLF6 | TF |
| ENSG00000155508 | POP2 | TF |
| ENSG00000172273 | MIZF | TF |
| ENSG00000133884 | DPF2 | TF |
| ENSG00000102038 | SNF2L1 | TF |
| ENSG00000032219 | ARID4A | TF |
| ENSG00000177463 | TR4 | TF |
| ENSG00000110925 | CSRNP2 | TF |
| ENSG00000257923 | CUX1 (p110) | TF |
| ENSG00000133740 | E2F5 | TF |
| ENSG00000165156 | ZHX1 | TF |
| ENSG00000109111 | SUPT6H | TF |
| ENSG00000148308 | TFIIIC63 | TF |
| ENSG00000144021 | Ciao 1 | TF |
| ENSG00000166402 | TUB | TF |
| ENSG00000165417 | TFIIA alpha/beta chains | TF |
| ENSG00000141568 | FOXK2 (ILF1-4) | TF |
| ENSG00000103168 | TAF1C | TF |
| ENSG00000067955 | CBF beta | TF |
| ENSG00000205250 | E2F4 | TF |
| ENSG00000196387 | ZNF140 | TF |
| ENSG00000186448 | VHLaK | TF |
| ENSG00000129691 | ASH2L | TF |
| ENSG00000153207 | ELYS | TF |
| ENSG00000166478 | ZNF143 | TF |
| ENSG00000170296 | GABARAP | Receptor |
| ENSG00000204580 | DDR1 | Receptor |
| ENSG00000142192 | APP | Receptor |
| ENSG00000148053 | TrkB | Receptor |
| ENSG00000140391 | Tetraspanin-3 | Receptor |
| ENSG00000135333 | Ephrin-A receptor 7 | Receptor |
| ENSG00000154134 | ROBO3 | Receptor |
| ENSG00000205336 | GPR56 | Receptor |
| ENSG00000077782 | FGFR1 | Receptor |
| ENSG00000092421 | Semaphorin 6A | Receptor |
| ENSG00000008952 | SEC62 | Receptor |
| ENSG00000187323 | Dcc | Receptor |
| ENSG00000196576 | Plexin B2 | Receptor |
| ENSG00000169855 | ROBO1 | Receptor |
| ENSG00000179915 | Neurexin 1-alpha | Receptor |
| ENSG00000121966 | CXCR4 | Receptor |
| ENSG00000113594 | LIFR | Receptor |
| ENSG00000153707 | PTPR-delta | Receptor |
| ENSG00000173482 | PTPR-mu | Receptor |
| ENSG00000164050 | Plexin B1 | Receptor |
| ENSG00000044524 | Ephrin-A receptor 3 | Receptor |
| ENSG00000178568 | ErbB4 | Receptor |
| ENSG00000106278 | PTPR-zeta | Receptor |
| ENSG00000132718 | Synaptotagmin XI | Receptor |
| ENSG00000118432 | CNR1 | Receptor |
| ENSG00000150471 | Lphn3 | Receptor |
| ENSG00000159461 | AMFR | Receptor |
| ENSG00000011454 | GPR21 | Receptor |
| ENSG00000138760 | CD36L2 | Receptor |
| ENSG00000205213 | GPR48 | Receptor |
| ENSG00000173726 | TOM20 | Receptor |
| ENSG00000204681 | GBR1 | Receptor |
| ENSG00000067141 | Neogenin | Receptor |
| ENSG00000105426 | PTPR-sigma | Receptor |
| ENSG00000167615 | LENG8 | Receptor |
| ENSG00000130827 | Plexin A3 | Receptor |
| ENSG00000142949 | PTPRF (LAR) | Receptor |
| ENSG00000150093 | ITGB1 | Receptor |
| ENSG00000068078 | FGFR3 | Receptor |
| ENSG00000140538 | TrkC | Receptor |
| ENSG00000196776 | CD47 | Receptor |
| ENSG00000116106 | Ephrin-A receptor 4 | Receptor |
| ENSG00000108679 | 90K | Receptor |
| ENSG00000140443 | IGF-1 receptor | Receptor |
| ENSG00000137872 | Semaphorin 6D | Receptor |
| ENSG00000067167 | TRAM1 | Receptor |
| ENSG00000163785 | RYK | Receptor |
| ENSG00000067715 | Synaptotagmin I | Receptor |
| ENSG00000132872 | Synaptotagmin IV | Receptor |
| ENSG00000072274 | TfR1 | Receptor |
| ENSG00000100442 | FKBP3 | Receptor |
| ENSG00000182168 | UNC5C | Receptor |
| ENSG00000185920 | PTCH1 | Receptor |
| ENSG00000119185 | ICAP-1 | Receptor |
| ENSG00000196277 | mGluR7 | Receptor |
| ENSG00000107771 | KIAA1128 | Receptor |
| ENSG00000063660 | Glypican-1 | Receptor |
| ENSG00000076356 | Plexin A2 | Receptor |
| ENSG00000099250 | Neuropilin-1 | Receptor |
| ENSG00000162512 | Syndecan-3 | Receptor |
| ENSG00000156298 | Tetraspanin-7 | Receptor |
| ENSG00000130227 | Exportin 7 | Receptor |
| ENSG00000154639 | CXADR | Receptor |
| ENSG00000185008 | ROBO2 | Receptor |
| ENSG00000145242 | Ephrin-A receptor 3 | Receptor |
| ENSG00000114354 | TFG | Receptor |
| ENSG00000115365 | LANCL1 | Receptor |
| ENSG00000100216 | TOM22 | Receptor |
| ENSG00000110076 | Neurexin-2 alpha | Receptor |
| ENSG00000157193 | APOER2 | Receptor |
| ENSG00000123384 | A2M receptor | Receptor |
| ENSG00000204217 | BMP receptor 2 | Receptor |
| ENSG00000144724 | PTPR-gamma | Receptor |
| ENSG00000139112 | GABARAPL1 | Receptor |
| ENSG00000163291 | PAQR3 | Receptor |
| ENSG00000072071 | LEC2 | Receptor |
| ENSG00000070018 | LRP6 | Receptor |
| ENSG00000064547 | LPA2 receptor | Receptor |
| ENSG00000148358 | Gpr107 | Receptor |
| ENSG00000133216 | Ephrin-B receptor 2 | Receptor |
| ENSG00000154174 | TOM70 | Receptor |
| ENSG00000114554 | Plexin A1 | Receptor |
| ENSG00000133019 | ACM3 | Receptor |
| ENSG00000134243 | Sortilin | Receptor |
| ENSG00000154928 | Ephrin-B receptor 1 | Receptor |
| ENSG00000136986 | Derlin1 | Receptor |
| ENSG00000168758 | Semaphorin 4C | Receptor |
