# Supplementary material for: Stabilization of HIF-1α and HIF-2α, up-regulation of MYCC and accumulation of stabilized p53 constitute hallmarks of CNS-PNET animal model
Source: PLoS One. 2017 Mar 1;12(3):e0173106. doi: 10.1371/journal.pone.0173106 (PMC5332108; doi:10.1371/journal.pone.0173106)
Supplement: S2 Table — (DOCX) [file pone.0173106.s005.docx]

**S2 Table**

| **Ab** | **LC25-RT** | **LC26-RT** | **LCAS-RT** |
| --- | --- | --- | --- |
| Ki-67 |  | X | X |
| Pou5F1 | X | X | X |
| Nestin | X | X | X |
| SOX2 | X | X | X |
| Vimentin |  |  | X |
| P53 | X | X | X |
| YB1 | X | X | X |
| MDM2 |  | X | X |
| MYCC | X | X | X |
| MYCC(S62) |  |  | X |
| MAX | X | X | X |
| HIF2a | X | X | X |
| HIF1a |  | X | X |
